# Contribution to the knowledge of the genus *Takobia* Novikova & Kluge, 1987 (Ephemeroptera, Baetidae) in Central Asia

**DOI:** 10.3897/zookeys.1071.71582

**Published:** 2021-11-18

**Authors:** Pavel Sroka, Zohar Yanai, Dmitry Palatov, Jean-Luc Gattolliat

**Affiliations:** 1 Biology Centre of the Czech Academy of Sciences, Institute of Entomology, Branišovská 31, 37005 České Budějovice, Czech Republic Biology Centre of the Czech Academy of Sciences, Institute of Entomology České Budějovice Czech Republic; 2 The Steinhardt Museum of Natural History, Tel Aviv University, Tel Aviv 6997801, Israel Tel Aviv University Tel Aviv Israel; 3 A. N. Severtsov Institute of Ecology and Evolution of RAS, 119071, Moscow, Russia A. N. Severtsov Institute of Ecology and Evolution of RAS Moscow Russia; 4 Musée cantonal de zoologie, Palais de Rumine, Place de la Riponne 6, 1014 Lausanne, Switzerland Musée cantonal de zoologie Lausanne Switzerland; 5 University of Lausanne (UNIL), Department of Ecology and Evolution, 1015 Lausanne, Switzerland University of Lausanne Lausanne Switzerland

**Keywords:** *Alainites*, mayflies, new species, *Nigrobaetis*, redescription, systematics, taxonomy

## Abstract

Based on the original type material, the nymphal stage of the mayfly *Takobiamaxillare* is redescribed; in parallel, a lectotype is designated. *Takobiamaxillare* is the type species of the genus *Takobia*, and an accurate and complete knowledge of its morphology is crucial to the delimitation of this problematic genus and clarification of its phylogenetic affinities. Ambiguous characters, previously reported for this species in the literature are clarified. Furthermore, two new species in the same genus are described, namely *Takobiasinusopalpata***sp. nov.** and *Takobiashughnonica***sp. nov.** based on the morphology of nymphs from Central Asia, supplemented with *COI* sequences. Implications for the systematics of *Takobia* and related taxa are discussed and the need for an extensive phylogenetic study of this group is stressed.

## Introduction

Baetidae encompass more than 1150 species nested in 115 genera, making it the most speciose family of all mayflies. Its systematics is accordingly complicated, and subject to frequent changes mainly related to generic concepts and species delimitation. Some taxa within Baetidae have particularly complex histories, such as *Nigrobaetis* Novikova & Kluge, 1987, *Alainites* Waltz & McCafferty, 1994, and *Takobia* Novikova & Kluge, 1987, which have all been subject to several synonymies and changes in rank between species groups, subgenera, and genera (Müller-Liebenau 1969; Novikova and Kluge 1987, 1994; Waltz et al. 1994; Waltz & McCafferty 1997; Jacob 2003; Kluge and Novikova 2014).

In this study, we focus on *Takobiamaxillare* (Braasch & Soldán, 1983), the type species of the problematic taxon *Takobia*. For any future extensive analysis aimed at assessing the relevance and extent of *Takobia*, detailed knowledge of its type species is of crucial importance. The species was originally described by Braasch and Soldán (1983) under the binomial combination “*Centroptilummaxillare*”. The description was based on 69 nymphs collected in Uzbekistan in 1980. Novikova and Kluge (1987) published a redescription of this species using material from Tajikistan and Kazakhstan, without studying the original type material from Braasch and Soldán. A description of the female imago was also provided for the first time by Novikova and Kluge (1987), associated with a nymph by rearing. These authors placed *C.maxillare* as a type species of the newly created subgenus Takobia Novikova & Kluge, 1987 within the genus *Baetis* Leach, 1815.

Subsequently, Novikova and Kluge (1994) redefined the delimitation of the genus *Baetis* in a wider sense to encompass three subgenera: *Baetis* s. s., *Labiobaetis* Novikova & Kluge, 1987, and *Nigrobaetis*. They claimed the latter was a senior synonym of *Takobia*, hence introducing a new combination entitled Baetis (Nigrobaetis) maxillaris (Braasch & Soldán, 1983). Some characters of the male imago were also reported for the first time in Novikova and Kluge (1994), based on reared material from Tajikistan. The authors also provided important details on nymphal habitats. The species was included in the key to freshwater invertebrates of Russia (Kluge 1997) as Baetis (Nigrobaetis) maxillaris. It is worth mentioning that the subgeneric delimitations suggested by Novikova and Kluge (1994) are no longer accepted, not even by their original proponents (Kluge and Novikova 2014, 2016).

Waltz et al. (1994) revalidated *Takobia* and raised it, together with *Nigrobaetis*, to the generic level. *Takobia* was still considered monospecific. In the same paper, Waltz et al. (1994) established the new genus *Alainites* for *Baetismuticus* (Linnaeus, 1758) and related species.

Kluge and Novikova (2014) synonymized the widely distributed and diversified genus *Alainites* with *Takobia*, thus considerably increasing the number of species in *Takobia*. Some subsequent authors used *Takobia* sensu Kluge and Novikova (2014) as a subgenus of *Nigrobaetis* (e.g., Martynov and Godunko 2017; Bojková et al. 2018). However, the synonymy of *Alainites* with *Takobia* was not generally followed and accepted, therefore many authors continued to use the genus *Alainites* (e.g., Gattolliat et al. 2015; Fujitani et al. 2017; Cruz et al. 2020). It is important to note that none of the proposals for the systematic treatment of the taxa mentioned above were based on cladistic analyses.

Since *T.maxillare* represents the type species of *Takobia*, an accurate knowledge of its morphology is a key prerequisite for the precise delimitation of the genus and is of significant importance for the generic attribution of all species of *Alainites* (also including species previously described in *Acerbaetis* Kang & Yang, 1994, in Kang et al. 1994, which was synonymized with *Alainites* by Waltz and McCafferty 1997). The original description and illustrations of *T.maxillare* are of reasonably good quality, however several characters important for assessing its relationship to other taxa within the *Alainites*/*Nigrobaetis*/*Takobia* complex have only been introduced in subsequent studies. Increasing uncertainty and confusion about *T.maxillare* morphology, inconsistencies on important morphological characters persist between the original description of Braasch and Soldán (1983) and the redescription of Novikova and Kluge (1987).

Therefore, the present study aims to provide an updated redescription of *T.maxillare* and analyze discrepancies between the descriptions of Braasch and Soldán (1983) and Novikova and Kluge (1987), based on the direct observation of the original type material. While searching for the fresh material of *T.maxillare* from Central Asia, we have discovered two new species closely related to *T.maxillare* and describe them herein. We also discuss the character distribution of these two species and compare them with *T.maxillare* and other members of the *Alainites*/*Nigrobaetis*/*Takobia* complex.

## Materials and methods

### Material examined

The original type material of *T.maxillare* was obtained from the collection of the Biology Centre of the Czech Academy of Sciences, Institute of Entomology, České Budějovice, Czech Republic (**IECA**), where it has been stored in ca. 70% ethanol in room temperature. It counts 164 nymphs (including lectotype) still deposited in IECA (159 in EtOH, 3 on slides, and 2 on SEM stubs) and 10 nymphs newly deposited in Museum of Zoology, Lausanne, Switzerland (**MZL**) (8 in alcohol, code GBIFCH 00829873; 2 used for DNA extraction (failed), codes GBIFCH00895419 and GBIFCH00895420). A comparative material of *Alainites* and *Nigrobaetis* species was obtained from IECA and MZL. The material of two new species described in this study was obtained by D. Palatov during several field trips to Central Asia between years 2012 and 2017, always in the period May-July. The nymphs were collected by kick sampling and after sorting subsequently stored in 96% ethanol in -20 °C. This material is deposited in IECA, MZL, and Zoological Museum of Moscow State University, Moscow (**ZMMU**) (for the number of specimens see the descriptions of individual species).

### Morphological study

Some specimens were mounted on slides with HydroMatrix (MicroTech Lab, Graz, Austria). Drawings were made using a stereomicroscope Leica M205 C and a microscope Olympus BX41, both equipped with a drawing attachment. Photographs were made using a Canon EOS 6D camera and the Visionary Digital Passport imaging system and processed with Adobe Photoshop Lightroom (http://www.adobe.com) and Helicon Focus version 5.3 (http://www.heliconsoft.com). Photographs were subsequently enhanced with Adobe Photoshop CS6. For scanning electron microscopy, samples were gradually transferred to acetone, critical point dried, and coated with gold by sputtering using a Baltec SCD050 Sputter Coater. Observations were made on the Jeol JSM 7401F at 4 kV scanning microscope at the Biology Centre CAS, České Budějovice, Czech Republic.

### Molecular study

DNA was extracted from two individuals per species of *Takobiamaxillare* (failed) and of the two newly described species. In addition, we extracted DNA for the first time from two additional species from central Asia: *Alainitestalasi* (Novikova & Kluge, 1994) and *A.kars* (Thomas & Kazancı, in Kazancı and Thomas 1989) (Table 1). Total genomic DNA was extracted using the BioSprint 96 extraction robot (Qiagen Inc., Hilden, Germany), following the supplier’s instructions. The non-destructive protocol described in Vuataz et al. (2011), which enables post-extraction morphological study of specimens, was implemented. We then amplified a 658-bp fragment at the 5’ end of the mitochondrial cytochrome c oxidase subunit I gene (COI), corresponding to the standard animal barcode region, using the HCO2198 and LCO1490 primers (Folmer et al. 1994). Polymerase Chain Reaction (PCR) was conducted in a volume of 33 μl, consisting of 5 μl of template DNA, 1.65 μl (10 μM) of each primer, 0.26 μl (25 mM) of dNTP solution (Promega), 6.6 μl of 10X buffer (Promega) containing 7.5 mM of MgCl_2_, 3.3 μl (25 mM) of MgCl_2_, 1 U of Taq polymerase (Promega), and 14.34 μl of sterile ddH_2_O. Optimized PCR conditions included initial denaturation at 95 °C for 5 min, 38 cycles of denaturation at 95 °C for 40 s, annealing at 50 °C for 40 s, and extension at 72 °C for 40 s, with final extension at 72 °C for 7 min. Purification and automated sequencing was carried out in Microsynth (Balgach, Switzerland).

**Table 1.** Taxa used for genetic distance analysis (mitochondrial COI sequences) with GenBank accession numbers (novel sequences are highlighted in bold font).

Species	GenBank accession numbers
*Takobiashughnonica* sp. nov. (paratype)	**MZ983793**, **MZ983794**
*Takobiasinusopalpata* sp. nov. (paratype)	**MZ983795**, **MZ983796**
*Alainitesalbinatii*	HG934994, HG934995
*Alainiteskars*	**MZ983797**, **MZ983798**
*Alainitesmuticus*	HG934999, JN299112
*Alainitestalasi*	**MZ983799**, **MZ983800**
*Alainitesyixianii*	GU479735
*Nigrobaetisbacillus*	MH823363, MH823364
*Nigrobaetisdigitatus*	JN164308, JN164309, LT626141
*Nigrobaetisgracilis*	JN164320
*Nigrobaetisminutus*	HM417038
*Nigrobaetisniger*	JN164310, JN164311, KC158570, KC158571
*Nigrobaetisparamakalyani*	LC056973
*Nigrobaetisvuatazi*	HE651544

Related taxa were added to the analysis, based on published sequences in GenBank database (https://www.ncbi.nlm.nih.gov/; see Table 1). Sequences were inspected and edited using Geneious Prime v. 2019.0.4 (Biomatters Ltd.) and pairwise distances calculated with MEGA-X v. 10.0.5 (Kumar et al. 2018) using a K2P model.

## Results

### Redescription of *Takobiamaxillare*

#### Takobia maxillare

Taxon classificationAnimaliaEphemeropteraBaetidae

(Braasch & Soldán, 1983)

C615D200-1E6F-5C11-9A49-DE22590F3564

Figs 1A, D ; 3 , 4

##### Differential diagnosis.

*Takobiamaxillare* can be easily separated from other related species by the combination of the following characters: 1) maxillary palp highly developed with the segment I widened apically and segment II straight; 2) labrum dorsally covered with numerous setae, none of them arranged in a row; 3) right prostheca reduced, apically bifid; 4) labial palp segment III quadrangular, asymmetrical, with a short projection lateroapically; 5) claw edentate, subequal to 1/2 of corresponding tarsus; 6) paraproct with a short prolongation bent ventrally.

##### Description of nymph.

**Length**. Female body 6.8–9.1 mm; cerci 4.6–5.4 mm; median caudal filament 3.4–4.5 mm; male body 5.0–7.2 mm; cerci 4.2–5.8 mm; median caudal filament 2.5–3.5 mm.

**Figure 1.**
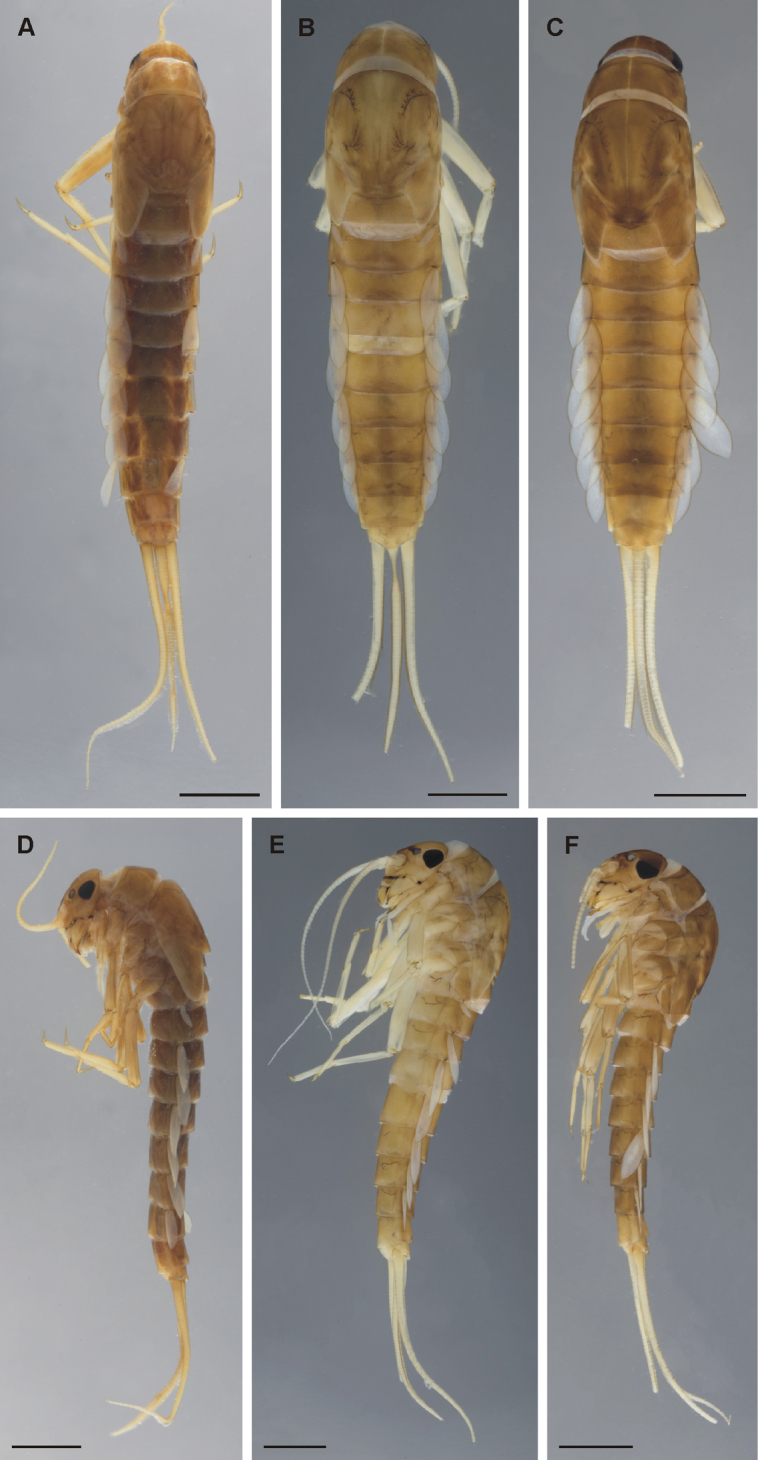
*Takobia* nymphs, habitus photographs **A***T.maxillare*, habitus in dorsal view **B***T.sinusopalpata* sp. nov., habitus in dorsal view **C***T.shughnonica* sp. nov., habitus in dorsal view **D***T.maxillare*, habitus in lateral view **E***T.sinusopalpata* sp. nov., habitus in lateral view **F***T.shughnonica* sp. nov., habitus in lateral view. Scale bar:1 mm.

**Coloration and texture**. General coloration brown (Fig. 1A, D). Head uniformly brown with vermiform marks visible on vertex and frons in some specimens. Turbinate eyes in male nymphs purple-brown. Legs ecru. Thorax brown with some areas of darker coloration. Abdominal tergites medium brown without any pattern. Abdominal sternites light brown. Cerci ecru to light brown without bands or pattern. Original coloration probably faded after more than 40 years of storage in alcohol. Surface of body shagreened, most pronounced on head capsule and thorax (Fig. 4C).

**Figure 2.**
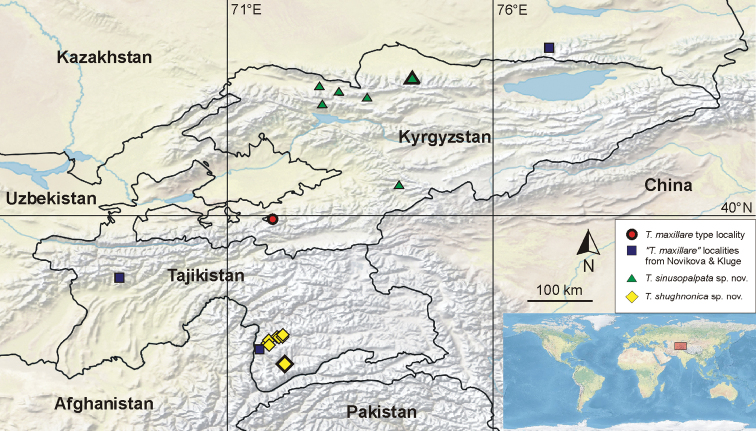
Map with known occurrences of individual *Takobia* species. Explanation of symbols directly in the figure. The larger symbol with thickened border indicates a type locality.

**Figure 3.**
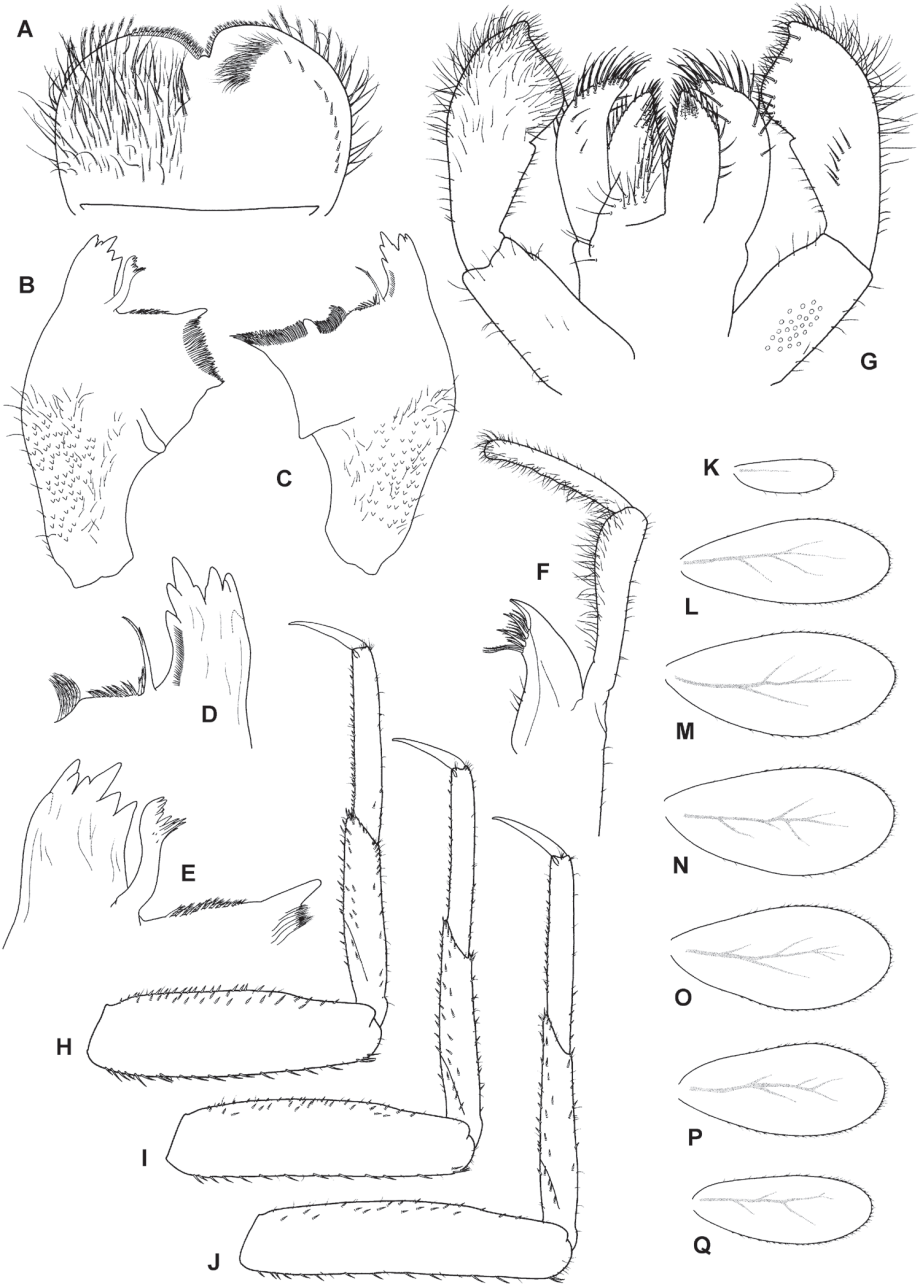
*T.maxillare*, nymph **A** labrum (left side dorsal view, right side ventral view) **B** left mandible (dorsal view) **C** right mandible (dorsal view) **D** right mandible, detail of incisors and prostheca (dorsal view) **E** left mandible (dorsal view), detail of incisors and prostheca (dorsal view) **F** maxilla (dorsal view) **G** labium (left side ventral view, right side dorsal view) **H** foreleg (dorsal view) **I** middle leg (dorsal view) **J** hind leg (dorsal view) **K–Q** gills.

**Figure 4.**
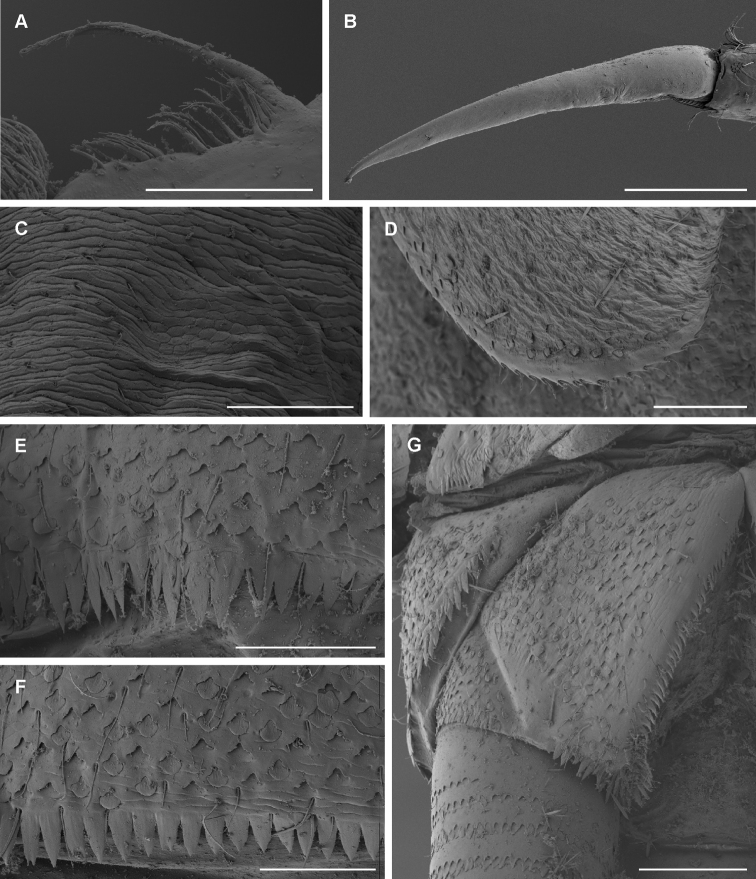
*T.maxillare*, nymph **A** detail of right prostheca **B** tarsal claw **C** pronotum texture **D** margin of gill plate V (dorsal view) **E** posterior margin of abdominal tergite V **F** posterior margin of abdominal sternite V **G** paraproct. Scale bars: 50 μm (**A, C–F**); 100 μm (**B, G**).

**Head**. Antennae close to each other, with a narrow interantennal carina; scape and pedicel with V-shaped scale insertions and sparse setae. Dorsal surface of labrum (Fig. 3A) evenly covered with numerous long setae and scattered small fine setae, distolateral arc of more prominent setae not distinguishable, almost no setae present along midline; ventral surface with row of ca. ten submarginal small, pointed setae laterally; distal margin fringed with ca. 12–17 short, followed by 8–12 long, feathered setae. Right mandible (Figs 3C, D; 4A) with sparse fine setae; incisors composed of eight pointed denticles (in nymphs long after molting, denticles become worn out and rounded), outer and inner incisor group with four denticles each; row of short fine setae along inner margin of incisors present; prostheca reduced and apically asymmetrically bifid (this bifurcation very inconspicuous, see Fig. 4A), slightly feathered; margin between prostheca and mola with tuft of fringed setae. Left mandible (Fig. 3B, E) with sparse fine setae; incisors composed of seven apically pointed denticles, outer and inner incisor groups not distinctly separated; prostheca with denticles and comb-shaped structure; margin between prostheca and mola with short, fringed setae. Hypopharynx apically covered with thin setae; lingua with central small protuberance; superlingua slightly longer than lingua. Maxilla (Fig. 3F) with incisors composed of three elongated and curved teeth; crown with two rows of setae, ventral one with small setae, dorsal row with three long stout dentisetae (apical dentiseta similar to maxillary teeth, relatively narrow); maxillary palp very long, nearly 3 × longer than galeolacinia, two-segmented, length of segment I nearly double length of galeolacinia, length of segment II subequal to segment I; segment I widened apically and slightly curved outward; segment II apically rounded; both segments with numerous thin setae, most dense along inner margin. Labium (Fig. 3G) with glossae subequal to paraglossae; both inner and outer margins of glossae with row of pointed setae, dorsal surface of glossae with well-defined group of fine setae subapically; ventral surface of glossae with group of long setae extending from basal part of glossa along its inner margin to apex; paraglossae with two rows of long, stout setae apically; labial palp three-segmented; segment I slightly shorter than segments II and III combined; segment II with very small medioapical protuberance and dorsal oblique row generally of six long setae; segment III asymmetrical, with medioapical part widely rounded and short projection lateroapically; all segments of labial palp with hair-like setae, present only occasionally on segments I and II, most dense on ventral surface of segment III; several distinct stout pointed setae present along apical part of segment III.

**Thorax**. Forelegs (Fig. 3H). Trochanter with ca. five marginal spine-like setae. Femur dorsally with one row of 16–18 medium, stout setae; additional dorsoapical setal patch formed by another 6–9 stout, medium setae; ventral margin with numerous stout, pointed short setae, some of these setae on lateral margin subparallel to ventral margin, villopore absent; lateral margin with occasional short hair-like setae and V-shaped scale bases with scales (not figured in Fig. 3H). Tibia with many setae along ventral margin and group of setae apically; scarce setae also elsewhere on surface of tibia and along dorsal margin; tibiopatellar suture present; lateral margins with scales and numerous scale bases. Tarsus with row of ca. 20 small, pointed setae on ventral margin; lateral margins with numerous scale bases. Tarsal claw (Fig. 4B) very slightly hooked, without any teeth, apical setae present, minute; length of tarsal claw subequal to 1/2 of tarsus; Mid and hindleg (Fig. 3I, J) similar to foreleg, except setae along ventral margin of femora less robust and more scarce in mid and hindlegs compared to forelegs. Hindwing pads present.

**Abdomen**. Tergites (Fig. 4E) not shagreened, with numerous V-shaped scale bases, scales (rounded apically), and thin hair-like setae; distal margin of tergite I with few occasional small triangular spines, tergites II–X with well-developed row of triangular spines (length:width ratio of spines in middle part of segment IV ca. 3:1–3:2); row of triangular spines on tergite X not interrupted in middle. Sternites (Fig. 4F) with scales, scale bases and setae similar to tergites; distal margin of sternites III–IX with row of triangular spines, this row interrupted in middle in sternite III and also interrupted in places of emerging gonostyli in male nymphs; triangular spines in lateral parts of sternite generally narrower than in central part of same sternite. Gills (Fig. 3K–Q) on segments I–VII, slightly asymmetrical, widened in distal portion, widely rounded apically; dorsal surface with scales and scale bases submarginally (Fig. 4D); tracheation faintly visible; margins serrated in distal 1/2, with row of fine setae; gill VII similar to gills II to VI. Paraproct (Fig. 4G) with abundant scales and scale bases (of same shape as on tergites and sternites); distinct prolongation bent dorsally; paraproct margin with ca. six or seven triangular spines laterally from prolongation and numerous slightly smaller spines medially from prolongation; prolongation margined with ca. ten elongated medium spines, with spines also on ventral surface; cercotractor with scales and scale bases, margin with triangular spines.

### Description of two new species of *Takobia*

#### Takobia sinusopalpata

Taxon classificationAnimaliaEphemeropteraBaetidae

Sroka & Gattolliat sp. nov.

BFA94836-7331-540F-8235-83E4AB97D929

http://zoobank.org/a0e1990c-fdaa-4a11-ac04-3c90d03ae1a2

Figs 1B, E ; 5 , 6

##### Material examined.

***Holotype***. mature male nymph (in EtOH): Kyrgyzstan, Chuy Region. Spring – left tributary of the Adygene Riv., 144 m a.s.l., 42°34.19'N, 74°28.57'E, 29.4.2016, Palatov leg., locality code: 17Kyrg. ***Paratypes***. 39 nymphs: same data as holotype (33 in EtOH, 2 on slides with HydroMatrix mounting medium, 2 on SEM stubs, 2 DNA voucher specimens). 1 nymph (in EtOH): Kyrgyzstan, Chuy Region, Korumdy Riv., 300 m upstream its mouth to Suusamyr Riv., 2214 m a.s.l., 42°12.40'N, 73°41.48'E, 1.5.2016, Palatov leg., locality code: 19Kyrg. 3 nymphs (in EtOH): Kyrgyzstan, Talas Region, Oshibulag Riv. – right tributary of Chychkan Riv., 1629 m a.s.l., 42°05.77'N, 72°48.19'E, 2.5.2016, Palatov leg., locality code: 25Kyrg. 1 nymph (in EtOH): Kyrgyzstan, Talas Region, Chon-chychkan Riv., ca. 1.5 km upstream Talas-Bishkek highway bridge, 1924 m a.s.l., 42°25.76'N, 72°44.03'E, 11.5.2016, Palatov leg., locality code: 60Kyrg. 12 nymphs (11 in EtOH, 1 on slide with HydroMatrix mounting medium): Kyrgyzstan, Talas Region. Otmek Riv. 2801 m a.s.l., 42°19.08'N, 73°05.77'E, 12.6.2016, Palatov leg., locality code: 65Kyrg. 6 nymphs (in EtOH): Kyrgyzstan, Osh Region, Kulun Riv., upstream from confluence of Kulaimende and Dungar Riv., 2229 m a.s.l., 40°30.46'N, 74°14.37'E, 1.5.2017, Palatov leg., locality code: 74 Kyrg.

Holotype and 30 paratypes are deposited in IECA, 5 paratypes including DNA voucher specimens are deposited in MZL, 27 paratypes are deposited in ZMMU. The inventory numbers for the MZL specimens are GBIFCH 00829874 for the specimens in alcohol, GBIFCH00895421 and GBIFCH00895422 for the specimens used for DNA extraction. GenBank accession numbers in Table 1.

##### Differential diagnosis.

*Takobiasinusopalpata* sp. nov. can be separated from other related species by the combination of the following characters: 1) maxillary palp highly developed with the segment I straight and segment II sinusoidal; 2) labrum dorsally covered with numerous setae, one central and two lateral forming the traditional disto-lateral arc of setae; 3) right prostheca reduced, basally bifid; 4) labial palp segment III quadrangular, slightly asymmetrical; 5) claw with one row of small teeth, subequal to 1/3 of corresponding tarsus; 6) paraproct with a short bent prolongation.

##### Description of nymph.

**Length**. Female body 6.8–7.4 mm; cerci and median caudal filament partially broken off, cerci assumed ca. 5 mm, medial caudal filament ca. 3.5 mm; male body 6.0–6.7 mm; cerci 4–5.2 mm; median caudal filament 2.6–3.4 mm.

**Coloration and texture**. General coloration brown (Fig. 1B, E). Head uniformly brown, darker between ocelli. Turbinate eyes in male nymphs brown. Legs ecru. Thorax dorsally brown without markings or pattern, thin pale longitudinal line medially. Abdominal tergites medium brown without any pattern. Abdominal sternites light brown. Gill plates whitish with dark margins. Cerci ecru to pale brown without bands or pattern. Surface of body indistinctly shagreened, most pronounced on head capsule and thorax (Fig. 6C).

**Figure 5.**
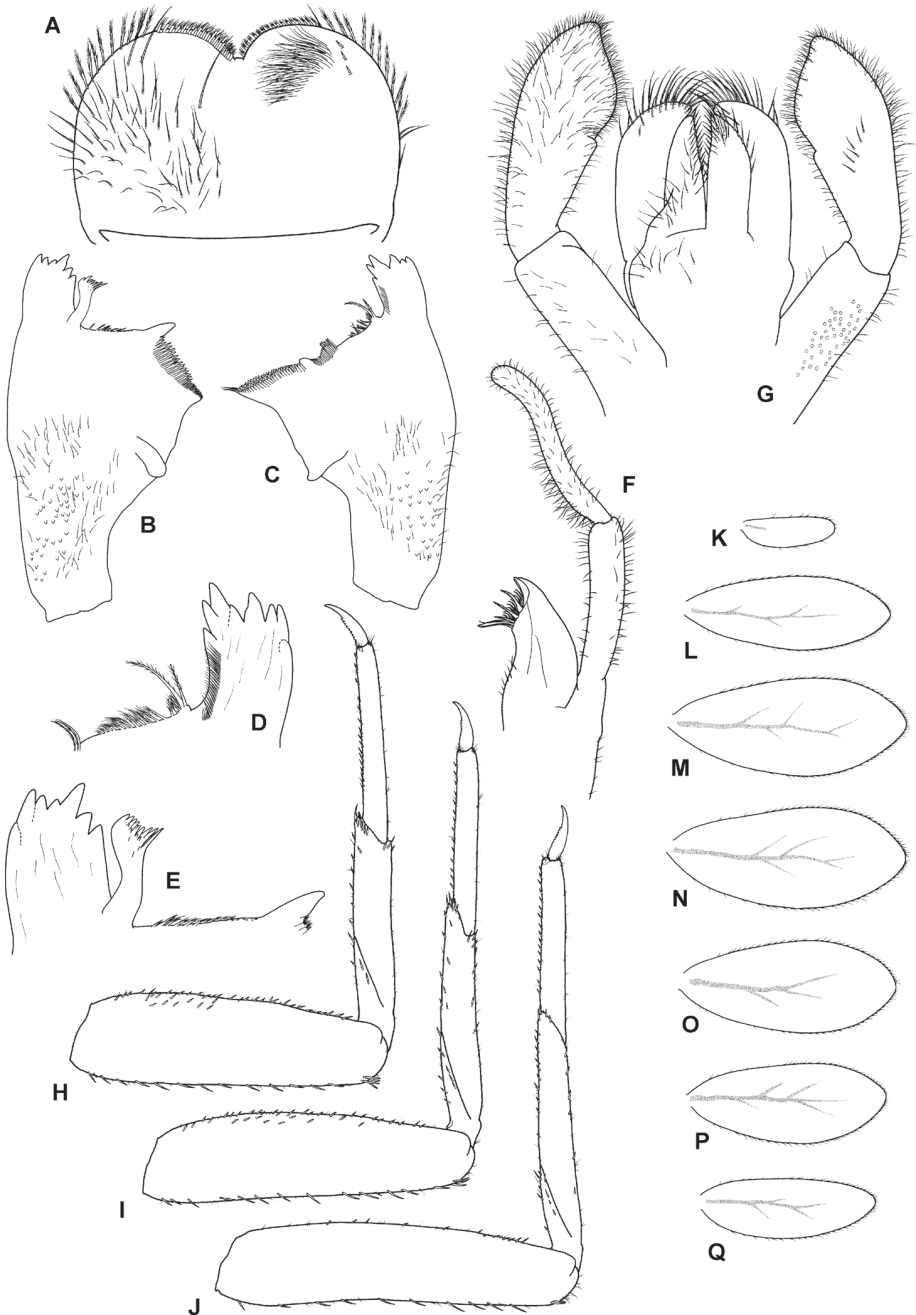
*Takobiasinusopalpata* sp. nov., nymph **A** labrum (left side dorsal view, right side ventral view) **B** left mandible (dorsal view) **C** right mandible (dorsal view) **D** right mandible, detail of incisors and prostheca (dorsal view) **E** left mandible (dorsal view), detail of incisors and prostheca (dorsal view) **F** maxilla (dorsal view) **G** labium (left side ventral view, right side dorsal view) **H** foreleg (dorsal view) **I** middle leg (dorsal view) **J** hind leg (dorsal view) **K–Q** gill plates.

**Figure 6.**
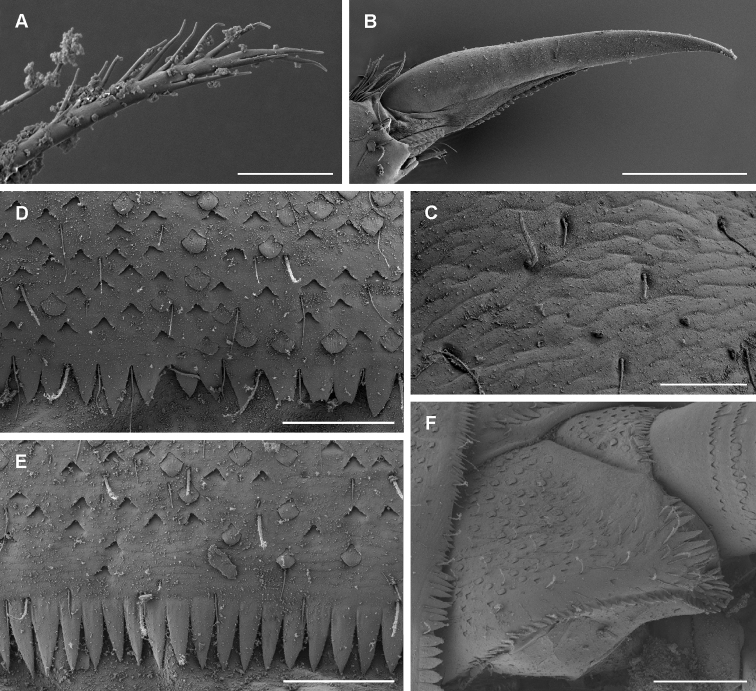
*Takobiasinusopalpata* sp. nov., nymph **A** detail of right prostheca **B** tarsal claw **C** pronotum texture **D** posterior margin of abdominal tergite V **E** posterior margin of abdominal sternite V **F** paraproct. Scale bars: 10 μm (**A**); 100 μm (**B, G**); 20 μm (**C**); 50 μm (**D, E**).

**Head**. Antennae close to each other, with a narrow interantennal carina; scape and pedicel with V-shaped scale insertions and sparse setae. Dorsal surface of labrum (Fig. 5A) covered with long setae and scattered small fine setae, in place of distolateral arc of prominent setae only one or two long setae, one prominent long seta submedially, almost no setae present along midline; ventral surface with short row of submarginal small, pointed setae laterally; distal margin fringed with ca. 17–21 short, followed by 8–12 long, feathered setae. Right mandible (Figs 5C, D; 6A) with sparse fine setae and scales dorsally in basal 1/2; incisors composed of eight apically pointed denticles (in nymphs long after molting, denticles become worn out and rounded), outer and inner incisor group with four denticles each; row of short fine setae along inner margin of incisors present; prostheca reduced and bifid, inserted on elevated projection, conspicuously feathered; margin between prostheca and mola with tuft of fringed setae. Left mandible (Fig. 5B, E) with sparse fine setae dorsally in basal 1/2; incisors composed of seven apically pointed denticles, outer and inner incisor group not distinctly separated; prostheca with denticles and comb-shaped structure; margin between prostheca and mola with short, fringed setae. Hypopharynx apically covered with thin setae; lingua with central small protuberance; superlingua of approximately same length as lingua. Maxilla (Fig. 5F) with incisors composed of three elongated and curved teeth; crown with two rows of setae, ventral one with only small setae, dorsal row with three long stout dentisetae (apical dentiseta similar to maxillary teeth, relatively broad); maxillary palp very long, ca. 2.7 × longer than galeolacinia, two-segmented, length of segment II approximately equal to segment I; segment I slightly curved inward, not distinctly widened apically; segment II sinusoidal, apically rounded; both segments with numerous thin setae, longest and most dense along inner margin of segment II in its basal 1/2. Labium (Fig. 5G) with glossae subequal to paraglossae; both inner and outer margins of glossae with row of pointed setae, dorsal surface of glossae with well-defined group of fine setae subapically; ventral surface of glossae with group of long setae extending from basal part of glossa along its inner margin to apex; paraglossae with two rows of long, stout setae apically; labial palp three-segmented; segment I slightly shorter than segments II and III combined; segment II with very small medioapical protuberance and dorsal oblique row of ca. 5–7 long setae; segment III elongated, asymmetrical, with medioapical part widely rounded and lateroapical part extended, with short indistinct projection; all segments of labial palp with hair-like setae, present only occasionally on segments I and II, most dense on ventral surface of segment III; several distinct stout pointed setae present along apical part of segment III.

**Thorax**. Forelegs (Fig. 5H). Trochanter with ca. six marginal spine-like setae. Femur dorsally with one row of ca. 13–16 medium, stout setae; additionally, dorsoapical setal patch formed by another 7–9 stout, medium setae; ventral margin with numerous stout, pointed short setae, some of these setae on lateral margin subparallel to ventral margin, villopore absent; lateral margin with occasional short hair-like setae and V-shaped scale bases with scales (not figured in Fig. 5H). Tibia with many setae along ventral margin and group of setae apically; scarce setae also elsewhere on surface of tibia and along dorsal margin; tibiopatellar suture present; lateral margins with scales and numerous scale bases. Tarsus with row of ca. 15–20 small, pointed setae on ventral margin; lateral margins with numerous scale bases. Tarsal claw (Fig. 6B) slightly hooked, with one row of ca. 10–16 small teeth, slightly increasing in size distally, apical setae present, very minute; length of tarsal claw ca. 1/3 of tarsus length; Mid and hindleg (Fig. 5I, J) similar to foreleg, except setae along ventral margin of femora, less robust and more scarce in mid and particularly hindlegs compared to forelegs. Hindwing pads present.

**Abdomen**. Tergites (Fig. 6D) not shagreened, with numerous V-shaped scale bases, scales (rounded apically), and thin hair-like setae; distal margin of tergite I without triangular spines, tergites II–X with well-developed row of triangular spines, slightly longer than wide; row of triangular spines on tergite X not interrupted in middle. Sternites with scales, scale bases and setae similar to tergites; distal margin of sternites IV–IX with row of long triangular spines, this row interrupted in middle in sternite IV and also interrupted in places of emerging gonostyli in male nymphs. Gills (Fig. 5K–Q) on segments I–VII, slightly asymmetrical, margins serrated mainly in distal 1/2, dorsal surface with scales and scale bases submarginally, tracheation faintly visible; gill I oval-shaped, rounded apically, ca. 3 × wider than long; gills II–VI widened in distal portion, narrowing and rounded apically, ca. 2.3–2.7 × wider than long; gill VII narrow, widened in middle portion, ca. 3 × wider than long. Paraproct (Fig. 6F) with abundant scales and scale bases (of same shape as on tergites and sternites); distinct prolongation bent dorsally; paraproct margin with ca. 5–10 triangular spines laterally from prolongation and numerous slightly smaller spines medially from prolongation; prolongation margined with ca. 10–20 elongated medium spines, with spines also on ventral surface; cercotractor with scales and scale bases, margin with triangular spines.

##### Etymology.

The name of the new species, *sinusopalpata*, refers to the sinusoidal shape of the second segment of the maxillary palps, very pronounced and characteristic for this species.

##### Distribution and ecology.

So far known from several localities in the Tien Shan Mountains (Kyrgyzstan). Nymphs were collected from stones and boulders sometimes covered with algae and moss in mountain springs, streams, and small rivers located at altitudes of 1600–2800 m a.s.l., at flow rates of 0.5–1.0 m/s, with water temperatures ca. 10–12°C (Fig. 9A, B).

#### Takobia shughnonica

Taxon classificationAnimaliaEphemeropteraBaetidae

Sroka & Gattolliat sp. nov.

C8405767-837C-5E88-A309-1A3C0C1C38DE

http://zoobank.org/2548add0-5f15-41f9-973f-8428e59e37f0

Figs 1C, F ; 7 , 8

##### Material examined.

***Holotype***. mature female nymph (in EtOH): Tajikistan, Roshtqal’a District. Spring near Sezhd village, 2966 m a.s.l., 37°12.65'N, 72°04.44'E, 2.7.2016, Palatov leg., locality code: 243Tj. ***Paratypes***. 39 nymphs (33 in EtOH, 2 on slides with HydroMatrix mounting medium, 2 on SEM stubs, 2 DNA voucher specimens): same data as holotype. 13 nymphs (in EtOH): Tajikistan, Shughnon District, unnamed river, right tributary of Gunt Riv., ca. 500 m S from Dehmiyona village, 2700 m a.s.l., 37°42.88'N, 71°53.61'E, 23.5.2012, Palatov leg., locality code: 15Tj. 28 nymphs (in EtOH): Tajikistan, Shughnon District. Vuzh-dara Riv., 3 km upstream Dehmiyona village, 2500 m a.s.l., 37°42.47'N, 71°57.29'E, 24.5.2012, Palatov leg., locality code: 31Tj. 2 nymphs (in EtOH): Tajikistan, Shughnon District, unnamed river, tributary of Gunt Riv. near Shitam village, 2500 m a.s.l., 37°44.30'N, 72°2.19'E, 31.5.2012, Palatov leg., locality code: 76Tj. 54 nymphs (in EtOH): Tajikistan, Shughnon District, stream on the slope of Gunt Riv. valley, ca. 3 km downstream from Ver village, 2875 m a.s.l., 37°43.27'N, 72°1.85'E, 5.6.2012, Palatov leg., locality code: 93Tj. 1 nymph (in EtOH): Tajikistan, Shughnon District, unnamed river near Tong village, 2480 m a.s.l., 37°35.78'N, 71°43.79'E, 8.6.2012, Palatov leg., locality code: 113Tj. 1 nymph (in EtOH): Tajikistan, Shughnon District, spring on slope of the Bogev-dara gorge. 2578 m a.s.l., 37°31.13'N, 71°41.98'E, 9.6.2012, Palatov leg., locality code: 120Tj. 17 nymphs (in EtOH): Tajikistan, Shughnon District, right source of the Bogev-dara Riv., 2928 m a.s.l., 37°29.89'N, 71°44.36'E, 10.6.2012, Palatov leg., locality code: 123Tj.

Holotype and 34 paratypes are deposited in IECA, 5 paratypes including DNA voucher specimens are deposited in MZL, 118 paratypes are deposited in ZMMU. The inventory numbers for the MZL specimens are GBIFCH 00829875 for the specimens in alcohol, GBIFCH00895421 and GBIFCH00895422 for the specimens used for DNA extraction. GenBank accession numbers in Table 1.

##### Differential diagnosis.

*Takobiashughnonica* sp. nov. can be separated from other related species by the combination of the following characters: 1) maxillary palp highly developed with the segment I straight and segment II slightly sinusoidal; 2) labrum dorsally covered with numerous setae, one central and two lateral forming the traditional disto-lateral arc of setae; 3) right prostheca reduced, basally bifid; 4) labial palp segment III symmetrical and almost conical; 5) claw with one row of teeth increasing in size toward the apex, subequal to 1/3 of corresponding tarsus; 6) paraproct with a short bent prolongation.

##### Description of nymph.

**Length**. Female body 6.4–7.4 mm; cerci 4.2–5.1 mm; median caudal filament 3.4–4.2 mm; male body 5.6–6.6 mm; cerci 3.5–3.6 mm; median caudal filament 2.7–3.0 mm.

**Coloration and texture**. General coloration brown (Fig. 1C, F). Head uniformly brown, darker in areas between compound eyes and between ocelli. Turbinate eyes in male nymphs dark brown. Legs light brown with patches of pale whitish color on lateral margin of femora. Thorax dorsally brown without markings or pattern, thin pale longitudinal line medially. Abdominal tergites I–VIII medium brown, lateral portions slightly paler. Tergites IX and X pale brown. In some specimens, two pale dots observable submedially on tergites VII and VIII. Abdominal sternites II–XIII light brown, sternite IX slightly paler, sternite I whitish. Gill plates whitish with dark margins. Cerci ecru to light brown without bands or pattern. Surface of body shagreened, most pronounced on head capsule and thorax (Fig. 8C).

**Figure 7.**
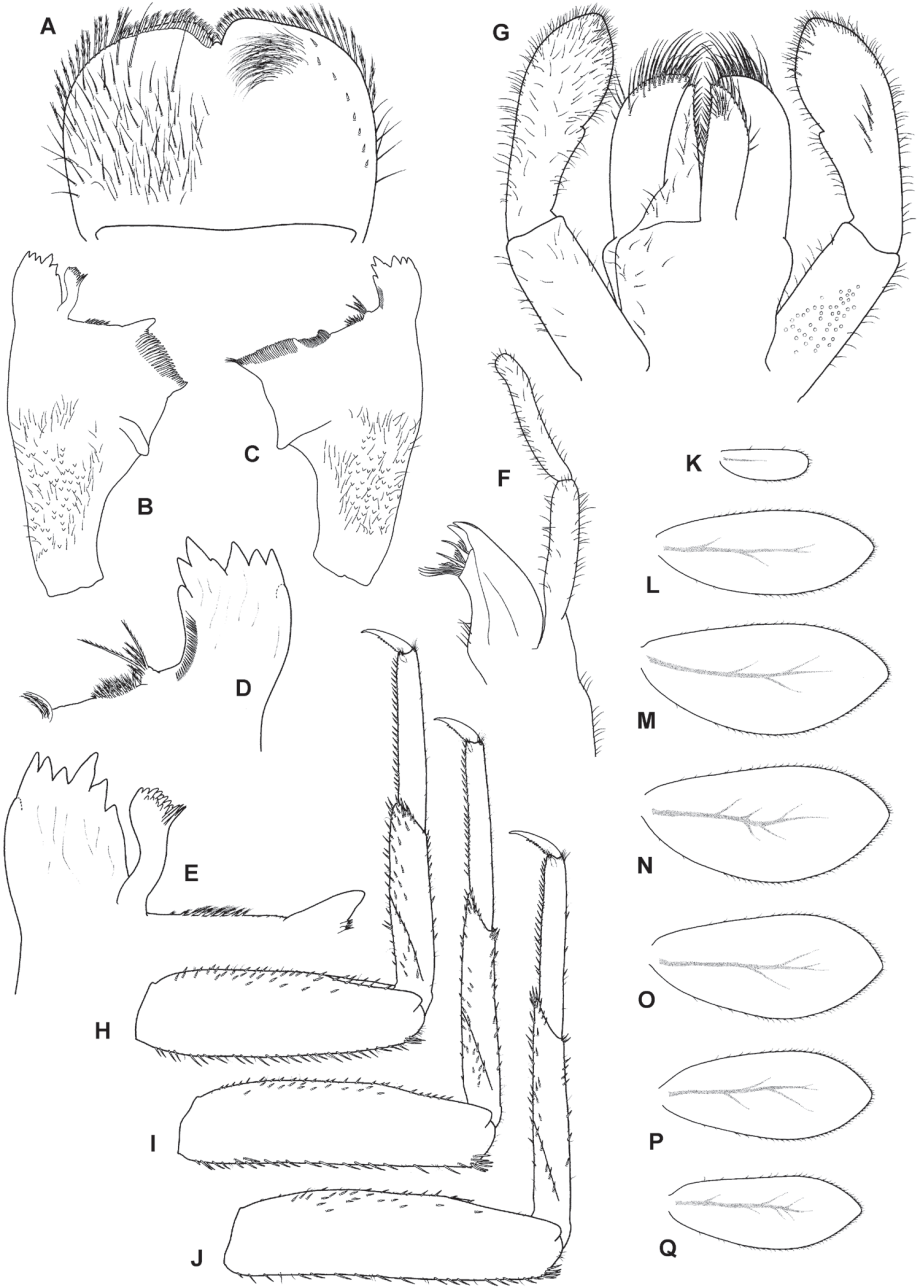
*Takobiashughnonica* sp. nov., nymph **A** labrum (left side dorsal view, right side ventral view) **B** left mandible (dorsal view) **C** right mandible (dorsal view) **D** right mandible, detail of incisors and prostheca (dorsal view) **E** left mandible, detail of incisors and prostheca (dorsal view) **F** maxilla (dorsal view) **G** labium (left side ventral view, right side dorsal view) **H** foreleg (dorsal view) **I** middle leg (dorsal view) **J** hind leg (dorsal view) **K–Q** gill plates.

**Figure 8.**
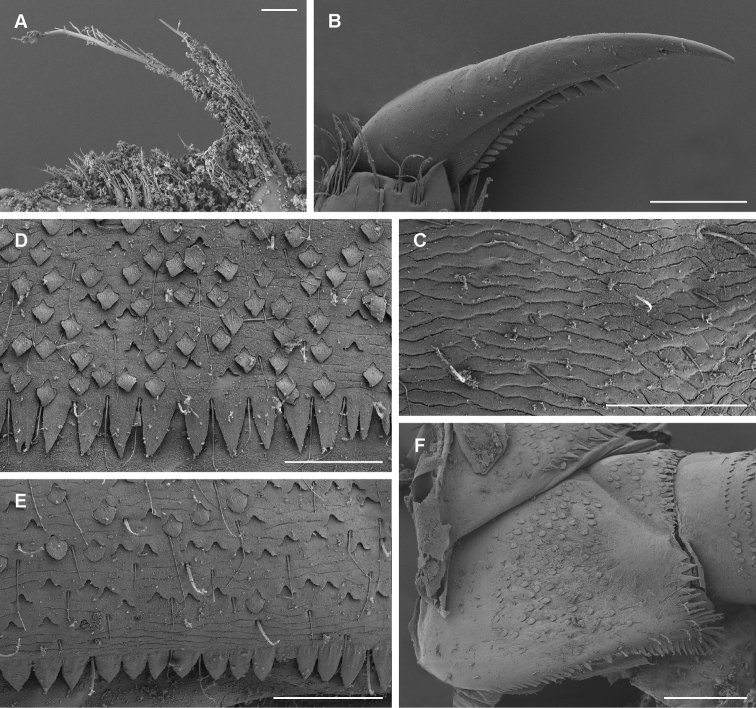
*Takobiashughnonica* sp. nov., nymph **A** detail of right prostheca **B** tarsal claw **C** pronotum texture **D** posterior margin of abdominal tergite VI **E** posterior margin of abdominal sternite VI **F** paraproct. Scale bars: 10 μm (**A**); 100 μm (**B, F**); 50 μm (**C–E**).

**Head**. Antennae close to each other, with a narrow interantennal carina; scape and pedicel with V-shaped scale insertions and sparse setae. Dorsal surface of labrum (Fig. 7A) evenly covered with numerous long setae and scattered small fine setae, in place of distolateral arc of prominent setae only two long setae, one promiment long seta submedially, almost no setae present along midline; ventral surface with row of submarginal small, pointed setae laterally; distal margin fringed with ca. 20–23 short, followed by 13–15 long, feathered setae. Right mandible (Figs 7C, D; 8A) with sparse fine setae; incisors composed of eight apically pointed denticles (in nymphs long after molting, denticles become worn out and rounded), outer and inner incisor group with four denticles each (outermost denticle of inner incisor group often worn out and indistinct); row of short fine setae along inner margin of incisors present; prostheca inserted on elevated projection, reduced, consisting of two prominent setae, accompanied by several shorter ones, all conspicuously feathered; margin between prostheca and mola with tuft of fringed setae. Left mandible (Fig. 7B, E) with sparse fine setae; incisors composed of seven apically rounded denticles, outer and inner incisor group not distinctly separated; prostheca with denticles and comb-shaped structure; margin between prostheca and mola with short, fringed setae. Hypopharynx apically covered with thin setae; lingua with central small protuberance; superlingua of approximately same length as lingua. Maxilla (Fig. 7F) with incisors composed of three elongated and curved teeth; crown with two rows of setae, ventral one with only small setae, dorsal row with three long stout dentisetae (apical dentiseta similar to maxillary teeth, relatively broad); maxillary palp very long, nearly 2 × longer than galeolacinia, two-segmented, length of segment II approximately equal to segment I; segment I straight, not distinctly widened apically; segment II slightly sinusoidal, apically rounded; both segments with numerous thin setae. Labium (Fig. 7G) with glossae subequal to paraglossae; both inner and outer margins of glossae with row of pointed setae, dorsal surface of glossae with well-defined group of fine setae subapically; ventral surface of glossae with group of long setae extending from basal part of glossa along its inner margin to apex; paraglossae with two rows of long, stout setae apically; labial palp three-segmented; segment I slightly shorter than segments II and III combined; segment II with very small medioapical protuberance and irregular dorsal oblique row of ca. seven or eight long setae; segment III symmetrical, elongated, narrowing towards apex, without any projection; all segments of labial palp with hair-like setae, present only sparsely on segments I and II, most dense on ventral surface of segment III; several distinct stout pointed setae present along inner margin of segment III.

**Thorax**. Forelegs (Fig. 7H). Trochanter with ca. six marginal spine-like setae. Femur dorsally with one row of 18–23 medium, stout setae; additionally, dorsoapical setal patch formed by another 7–9 stout, medium setae; ventral margin with numerous stout, pointed short setae, some of these setae on lateral margin subparallel to ventral margin, villopore absent; lateral margin with occasional short hair-like setae and V-shaped scale bases with scales (not figured in Fig. 7H). Tibia with many setae along ventral margin and group of setae apically; fewer setae also elsewhere on surface of tibiae and along dorsal margin; tibiopatellar suture present; lateral margins with scales and numerous scale bases. Tarsus with row of ca. 25–30 small, pointed setae on ventral margin; lateral margins with numerous scale bases. Tarsal claw (Fig. 8B) hooked, with single row of 12–15 well developed teeth, increasing in size distally; apical setae present, very minute; length of tarsal claw ca. 1/3 of tarsus length; Mid and hindleg (Fig. 7I, J) similar to foreleg, except setae along ventral margin of femora, less robust and more scarce in mid and hindlegs compared to forelegs. Hindwing pads present.

**Abdomen**. Tergites (Fig. 8D) slightly shagreened, with numerous V-shaped scale bases, scales (rounded apically), and thin hair-like setae; distal margin of tergite I without triangular spines, tergites II–X with well-developed row of triangular spines, slightly longer than wide; row of triangular spines on tergite X not interrupted in middle. Sternites with scales, scale bases and setae similar to tergites; distal margin of sternites IV–IX with row of triangular spines, this row interrupted in middle in sternite IV and also interrupted in places of emerging gonostyli in male nymphs; triangular spines in lateral parts of sternite generally narrower than in central part of same sternite. Gills (Fig. 7K–Q) on segments I–VII, slightly asymmetrical, margins serrated mainly in distal 1/2, dorsal surface with scales and scale bases submarginally, tracheation faintly visible; gill I oval-shaped, rounded apically, ca. 3 × wider than long; gills II to VII widened in distal portion, pointed apically, ca. 2.1–2.6 × wider than long. Paraproct (Fig. 8F) with abundant scales and scale bases (of same shape as on tergites and sternites); distinct prolongation bent dorsally; paraproct margin with ca. 3–5 triangular spines laterally from prolongation and numerous slightly smaller spines medially from prolongation; prolongation margined with ca. 15 elongated medium spines, with only minor spines on ventral surface; cercotractor with scales and scale bases, margin with triangular spines.

##### Etymology.

The species is named *shughnonica* after the local ethnicity and the historical region of Shughnon, where the species was discovered.

##### Distribution and ecology.

**Figure 9.**
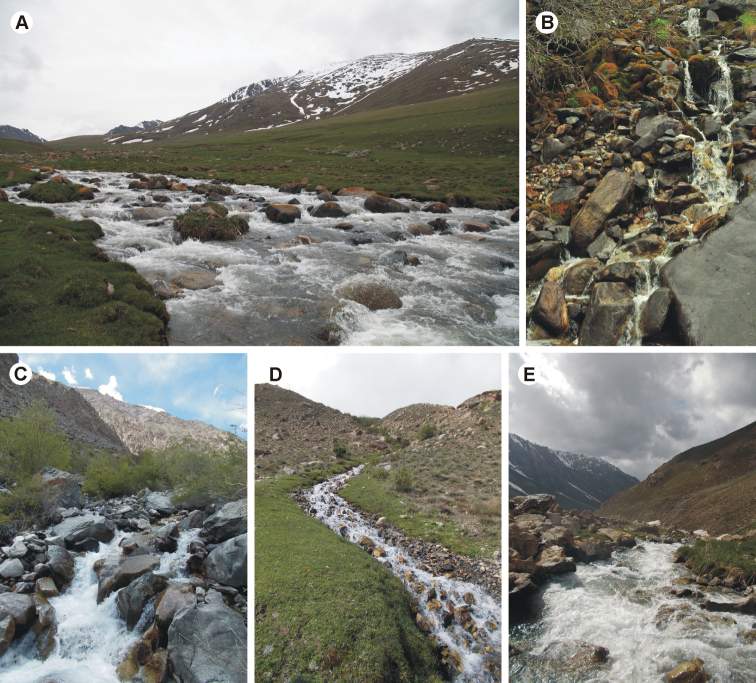
*Takobia* spp., examples of habitats **A** locality of *T.sinusopalpata* sp. nov. (Otmek Riv., locality code: 65Kyrg) **B** type locality of *T.sinusopalpata* sp. nov. (left tributary of the Adygene Riv., code: 17Kyrg) **C** locality of *T.shughnonica* sp. nov. (right tributary of Gunt Riv., code: 15Tj) **D** locality of *T.shughnonica* sp. nov. (stream on the slope of Gunt Riv. valley, code: 93Tj) **E** locality of *T.shughnonica* sp. nov. (right source of the Bogev-dara Riv., code: 123Tj).

So far known from several localities in the Pamir Mountains (Tajikistan). Nymphs were collected from stones and boulders sometimes covered with algae and moss in mountain springs and streams located at altitudes of 2480–2928 m a.s.l., at flow rates of 0.5–1.0 m/s, with water temperatures ca. 10–12°C (Fig. 9C–E).

### Molecular results

The monophyly of the two new *Takobia* species, as well as that of *A.talasi* and *A.kars*, were confirmed (Table 2): each of these species exhibited intra-specific similarities of 0–0.1%. The COI sequences of the two *Takobia* species are 13.5% different, confirming a close sibling relationship, yet far enough to be considered independent species. Between species in other genera, our analysis yielded a minimum distance of 7.5%, and usually much higher (> 20%). Our table contains a few problematic results. On the one hand, a few inter-specific distances lower than expected may be due to misidentification of the GenBank samples, which are not available to us for morphological re-examination. On the other hand, intra-specific distances higher than expected may be explained by taxa representing complexes of cryptic species, as shown by Sroka (2012) for *A.muticus*. These species belong to *Alainites* and *Nigrobaetis*, and are out of the scope of the present study.

**Table 2.** Kimura 2 parameter distance among sequences of the mitochondrial COI gene of selected *Takobia*, *Alainites*, and *Nigrobaetis* species (presenting mean and min–max distance for each group with >1 individual; for number of samples per each taxon see Table 1).

		**1**	**2**	**3**	**4**	**5**	**6**	**7**	**8**	**9**	**10**	**11**	**12**	**13**	**14**
**1**	*T.shughnonica*	0.0													
**2**	*T.sinusopalpata*	13.5 (13.5–13.5)	0.0												
**3**	*A.albinatii*	22.5 (22.2–22.8)	22.0 (21.9–22.1)	1.5											
**4**	*A.kars*	22.8 (22.7–22.9)	23.2 (23.1–23.3)	20.1 (19.9–20.3)	0.1										
**5**	*A.muticus*	25.0 (24.2–25.7)	24.8 (24.7–24.9)	17.0 (16.6–17.5)	22.3 (22.1–22.5)	17.4									
**6**	*A.talasi*	25.4 (25.4–25.4)	23.1 (23.1–23.1)	27.3 (27.1–27.6)	24.9 (24.8–25.0)	26.0 (24.3–27.8)	0.0								
**7**	*A.yixianii*	25.9 (25.9–25.9)	24.7 (24.7–24.7)	20.8 (20.5–21.1)	20.3 (20.2–20.4)	23.7 (23.1–24.3)	24.2 (24.2–24.2)	NA							
**8**	*N.bacillus*	26.6 (26.3–26.8)	26.1 (25.9–26.3)	22.3 (21.8–22.8)	22.1 (21.7–22.5)	25.4 (24.1–26.8)	24.9 (24.6–25.3)	0.8 (0.5–1.1)	0.6						
**9**	*N.digitatus*	22.5 (21.4–24.4)	22.8 (22.1–24.2)	23.3 (22.6–24.0)	24.2 (23.9–24.6)	25.3 (23.4–27.6)	24.6 (24.3–25.1)	23.5 (21.8–24.5)	24.4 (21.9–26.1)	9.9 (0.6–14.6)					
**10**	*N.gracilis*	28.3 (28.3–28.3)	27.7 (27.7–27.7)	23.6 (23.6–23.6)	25.1 (25.0–25.3)	25.1 (24.7–25.4)	24.8 (24.8–24.8)	25.4	26.5 (26.0–27.0)	24.5 (24.2–25.2)	NA				
**11**	*N.minutus*	25.1 (25.1–25.1)	22.5 (22.5–22.5)	25.5 (25.0–26.1)	24.5 (24.4–24.6)	27.1 (26.7–27.5)	25.4 (25.4–25.4)	24.3	25.7 (25.4–26.1)	23.9 (23.6–24.6)	21.8	NA			
**12**	*N.niger*	26.7 (25.8–27.6)	25.5 (24.8–26.4)	23.1 (22.0–24.2)	22.0 (21.6–22.4)	24.6 (23.4–25.8)	24.9 (24.3–25.7)	22.8 (22.0–23.0)	24.6 (23.2–25.8)	20.2 (19.4–21.3)	23.2 (22.4–24.8)	24.1 (22.9–25.3)	1.0 (0.5–1.9)		
**13**	*N.paramakalyani*	25.5 (25.5–25.5)	24.1 (24.1–24.1)	26.5 (25.9–27.1)	25.0 (24.9–25.1)	27.3 (27.2–27.5)	28.0 (28.0–28.0)	26.8	28.0 (27.7–28.4)	24.5 (22.8–25.3)	18.7	20.2	24.7 (24.3–25.5)	NA	
**14**	*N.vuatazi*	24.8 (24.8–24.8)	23.6 (23.6–23.6)	24.4 (24.1–24.6)	22.7 (22.6–22.9)	28.0 (27.7–28.4)	24.0 (24.0–24.0)	23.7	25.4 (25.1–25.0)	21.9 (21.4–22.3)	7.5	18.1	23.8 (22.9–24.4)	17.8	NA

## Discussion

### Remarks on the *T.maxillare* type material

The original type series consisted of nymphal material collected on a single locality (“Uzbekische SSR, Kuk-kul-See, S von Fergana, 20.5.1980, leg. T. SOLDÁN et M. TONNER”, as given in Braasch and Soldán 1983). The authors specified the existence of the holotype and 68 paratypes, split between the collections of T. Soldán in Prague (Czechia) and D. Braasch in Potsdam (Germany). The exact number of paratypes deposited in each collection was not specified in the original description, it is supposed that the holotype was most likely in Prague (“Typen in der Coll. SOLDÁN, Prag, Paratypen in der Coll. BRAASCH, Potsdam.“ in Braasch and Soldán 1983).

The collection of Dietrich Braasch is now housed mostly in the Stuttgart State Museum of Natural History, Germany, and partially in the Natural History Museum Potsdam and Senckenberg German Entomological Institute, Müncheberg, Germany. According to our inquiry to the curators of all these collections, there is no material of *T.maxillare* in any of them. Thus, either the types from D. Braasch should be considered lost or the type material was never split and all the types remained in T. Soldán’s collection.

The collection of Tomáš Soldán is now housed in the Biology Centre CAS, Institute of Entomology (IECA). There is material identified as *T.maxillare*, morphologically identical to the original description and labeled with the corresponding locality and the date of collection. However, there is no unambiguous label designating holotype and/or paratypes. Furthermore, the number of nymphs does not correspond to Braasch and Soldán (1983), since the vial contains 174 individuals instead of 69, as specified in the original description. There are no microscopic slides preserved.

### Type locality

The exact location of the *T.maxillare* type locality is unclear. The original publication specifies a lake in Uzbekistan, south of the town Fergana. However, the border with Kyrgyzstan is ca. 20 km south of Fergana (Farg’ona), and there is no substantial water body south of Fergana within the main territory of Uzbekistan. Nevertheless, there is a small exclave of Uzbekistan within Kyrgyzstan further south. Two lakes are situated nearby, although not directly within the exclave, but a few hundred meters past the border in Kyrgyzstan. The borders have however shifted compared to where they were in the 1980s when the lakes were administratively located in Uzbekistan. Soldán and Tonner, the collectors of the original material, probably would not have realized that they had crossed the administrative border anyway, being technically still within the USSR in 1980.

The above-mentioned lakes are named Qurbonko’l and Ko’kko’l in Kyrgyzian sources, Курбан-Кёль (Kurban-Kiol’) and Кок- Кёль (Kok-Kiol’) in Russian, although exact transliteration varies. We are convinced that “Kuk-Kul” in the original publication is a version of Ko’kko’l and the present location in Kyrgyzstan instead of Uzbekistan is caused by the close proximity to the Uzbekistan exclave and the recent changes in the administrative borders in the area. Thus, we define the type locality of *Takobiamaxillare* as follows: Kyrgyzstan, Ko’kko’l Lake, near border with Uzbekistan, Shakhimardan (Shohimardon) town, S of Fergana (Farg’ona), 39°56.10'N, 71°51.00'E.

### Lectotype designation

Based on the identical locality, morphology, and deposition of the material and despite the lack of proper labeling, we consider the material located in T. Soldán’s former collection as the type material of *T.maxillare*; all the specimens constitute syntypes according to ICZN Article 73.2. To ensure the stability of the species, we thus designate a lectotype (female mature nymph) and paralectotypes (173 nymphs, same data as lectotype) according to the ICZN Article 74. The material was collected by T. Soldán and M. Tonner on 20.5.1980 in the type locality specified above. For the deposition of the material, see the chapter “Material examined”.

### Amendment to the morphology of *T.maxillare*

When comparing the original description of *T.maxillare* by Braasch and Soldán (1983) with the type material, all morphological characters are congruent. However, the redescription published four years later (Novikova and Kluge 1987), exhibited multiple inconsistencies with both the original description, and the type material itself.

A very distinctive feature of *T.maxillare* is the elongated maxillary palps with the first segment widened apically and the second segment distinctly narrow in diameter (Fig. 3F; Braasch and Soldán 1983: fig. 15). This unique feature is visible even without the need to prepare a slide. In Novikova and Kluge (1987), the first segment of maxillary palp is not distinctly widened apically, and the second segment is even wider in diameter than the first (Novikova and Kluge 1987: fig. 2). The shape of the prostheca also slightly differs, although in this case it might be the result of variability or the limited visibility of some structures. The left prostheca is slightly wider apically in Novikova and Kluge (1987) than in Braasch and Soldán (1983). In the right prostheca, apical bifurcation is present in Braasch and Soldán (1983: fig. 14) and absent in Novikova and Kluge (1987: fig. 2). Our observations confirm the characters as depicted in Braasch and Soldán 1983 (Figs 3C, D, 4A). However, the right prostheca is bifurcated only in the apical part, which is sometimes hardly recognizable, only seen with certainty by using SEM (Fig. 4A). One more discrepancy occurs in the mouthparts in the shape of the labial palp, with the apical projection of the third segment being much broader in Novikova and Kluge (1987: fig. 2) than in Braasch and Soldán (1983: fig. 16) and the type material we have investigated (Fig. 3G).

In the type material of *T.maxillare*, the claws are apparently longer compared to the claws depicted in Novikova and Kluge (1987: fig. 2); in the types the tarsus is 2.3–2.5 × longer than the claw, whereas this ratio is 2.9 according to the illustration in Novikova and Kluge (1987: fig. 2). Scales feathered apically, documented on various body parts by Novikova and Kluge (1987: fig. 2), are actually not present on the type material of *T.maxillare* at all; these scales are instead smoothly rounded apically (Fig. 4D–G). The rectangular shape of scale sockets presented by Novikova and Kluge (1987: fig. 2) probably does not represent the true shape of the sockets, but rather the shape of scales themselves, since the shape of the scale base inserted inside a socket is often prominent to the eye when observed under a light microscope. This is identical in the *T.maxillare* type material.

The shape of the gills is also different between Novikova and Kluge (1987: fig. 2) and Braasch and Soldán (1983: fig. 16). In Novikova and Kluge (1987), gills II–VII are bluntly pointed apically, with the widest part at ca. 1/2 of the respective plate length. In the *T.maxillare* type material, these plates are more widely rounded apically, with the widest part at ca. 2/3 of the plate length (Fig. 3L–Q). This is consistent with the drawing of Braasch and Soldán (1983: fig. 22). Gill I in Novikova and Kluge (1987) is widened basally, which also does not correspond with the original description and the type material (Fig. 3K). The shape of paraproct is mostly similar in Novikova and Kluge (1987: fig. 2) and Braasch and Soldán (1983: fig. 20). The posteromedial extension is bent dorsally, thus not immediately visible from the ventral view.

Based on the characters compared above, we are rather confident that Novikova and Kluge (1987) actually depicted a different species than *T.maxillare*. It probably represents an additional undescribed species occurring in Central Asia, sharing some diagnostic characters with *T.maxillare*, although distinguishable on a specific level. This misidentification may have been derived from the uniquely long maxillary palp, which caused Novikova and Kluge (1987) to confuse their material with the only locally described species to share such a character. Consequently, the male and female imagines assigned to *T.maxillare* possibly do not actually belong to *T.maxillare*. The adult females were described by Novikova and Kluge (1987), reared from nymphs with morphology presumably specified in the species’ redescription, presented in the same paper (and as demonstrated above, different from *T.maxillare* types). The adult males were described later in Novikova and Kluge (1994), also based on reared material, and possibly from the same nymphal morphotype. The conspecificity of the adult stage of “*T.maxillare*” and a possible undescribed related nymph from the area remain to be tested in the future either by new rearing or DNA comparison.

### Distinctive morphological characters of *T.maxillare*, *T.sinusopalpata* sp. nov., and *T.shughnonica* sp. nov.

These three species possess a distinctive paraproct with a short bent prolongation. The paraproct presents similar but more pronounced prolongation in various species historically assigned to *Alainites* (Gattolliat 2011; Zrelli et al. 2012). A similar projection is exceptional in other lineages; it also occurs in *Indobaetis* Müller-Liebenau & Morihara, 1982 and *Papuanatula* Lugo-Ortiz & McCafferty, 1999, although its structure is different in these two taxa, and varies among species (Kluge and Novikova 2014).

The elongated maxillary palp is a character shared by *T.maxillare*, *T.sinusopalpata* sp. nov., and *T.shughnonica* sp. nov., and is much less developed in the various species assigned to *Alainites*. The shape of the palp significantly differs between these three species: in *T.maxillare*, the first segment is widened apically and curved outwards, while it is almost straight in *T.sinusopalpata* sp. nov. and *T.shughnonica* sp. nov.; the second segment is sinusoidal in *T.sinusopalpata* sp. nov., slightly sinusoidal in *T.shughnonica* sp. nov., and straight in *T.maxillare*.

It seems that the nymphal morphology of *T.sinusopalpata* sp. nov. and *T.shughnonica* sp. nov. is somewhat intermediary between *Alainites* sensu Waltz et al. (1994) (with type species *A.muticus*) and *Takobia* (with type species *T.maxillare*). Both new species exhibit a combination of characters partially similar to *T.maxillare*, notably sharing the elongated maxillary palps. On the other hand, both species possess denticles on the tarsal claws, contrary to *T.maxillare* and the undescribed *Takobia* species illustrated by Novikova and Kluge (1987), which exhibit tarsal claws devoid of even minute denticles. It is worth mentioning that in *T.sinusopalpata* sp. nov., the claw is only slightly curved, more distinctly elongated and the denticles are very small, more closely resembling the situation in *T.maxillare* than in the case of *T.shughnonica* sp. nov.

The presence of claw denticles is considered as a plesiomorphic condition in Baetidae, being subject to reduction in several non-related lineages. On the other hand, the elongated maxillary palp is almost unique within Baetidae and probably represents a synapomorphy of *T.maxillare*, *T.sinusopalpata* sp. nov., and *T.shughnonica* sp. nov. Therefore, we assign both new species described herein to the genus *Takobia*, primarily based on this character. The three species are also closely distributed geographically, therefore they may form a single lineage restricted to Central Asia.

### Synonymy of *Alainites* with *Takobia*

In the recent Baetidae phylogeny by Cruz et al. (2020), *Alainites* and *Takobia* were recovered as sister lineages, nesting within the same clade as *Nigrobaetis*, *Fallceon*, and *Caribaetis*. Kluge and Novikova (2014) suggested the synonymy of *Alainites* with *Takobia*. These authors argued that *T.maxillare* is a species with a unique morphology, phylogenetically clustering within *Alainites*, but exhibiting several apomorphies within the lineage, such as elongated maxillary palps and secondarily reduced claw denticles. The remaining representatives of *Alainites* are defined only by plesiomorphies with regard to *T.maxillare*. Since *T.maxillare* is only a single aberrant species within the lineage, using a generic name is redundant to distinguish a single apomorphic species; it should be classified in the same taxon with its plesiomorphic relatives instead. And since the genus *Takobia* is senior to *Alainites*, all *Alainites* species should be reclassified into *Takobia*.

However, the inconsistencies in the description of *T.maxillare* and later redescription by Novikova and Kluge (1987) point to the existence of more species in Central Asia with the same apomorphies as *T.maxillare*, as demonstrated above. This implication is further corroborated by our description of two more species exhibiting elongated maxillary palp closely resembling *T.maxillare*. More undescribed species similar to *T.maxillare* possibly occur in the Himalayas (unpublished data). There is likely a monophyletic lineage comprised of *T.maxillare* and all these species. The relationship of this lineage to *Alainites* is not clear at present. It might represent a lineage inside *Alainites*, rendering this genus paraphyletic and justifying the synonymy of *Alainites* with *Takobia* as suggested by Kluge and Novikova (2014). However, *Takobia* might also well constitute a sister lineage to *Alainites*. Thus, we prefer to consider *Takobia* and *Alainites* as separate genera, since *Takobia* comprises several derived species defined by a common apomorphic characters.

In conclusion, our results prove the existence of several Central Asian mayfly species closely related to *T.maxillare*. Two of them are newly described herein and another one erroneously assigned to *T.maxillare* in the literature. This was tested by the study of the original type material. The fact that *Takobia* hitherto consisted of a single species was only a consequence of our poor knowledge of the Central Asian mayfly fauna rather than *T.maxillare* really being something unique. The reclassification of all *Alainites* species based on such a premise is undesirable. Therefore, we refrain for the moment to follow the nomenclatural changes proposed by Kluge and Novikova (2014). We are convinced that any newly proposed classification of the *Alainites*/*Nigrobaetis*/*Takobia* complex must be based on a global phylogenetic analysis. Our study highlights the need for such an analysis and forms one of the necessary preliminary steps for the accomplishment of such a task.

## Supplementary Material

XML Treatment for Takobia maxillare

XML Treatment for Takobia sinusopalpata

XML Treatment for Takobia shughnonica

## References

[B1] Bojková J Sroka P Soldán T Namin JI Staniczek AH Polášek M Hrivniak Ľ Abdoli A Godunko RJ (2018) Initial commented checklist of Iranian mayflies, with new area records and description of *Procloeoncaspicum* sp. n. (Insecta, Ephemeroptera, Baetidae). ZooKeys 749: 87–123. 10.3897/zookeys.749.24104 PMC5904425 29674922

[B2] Braasch D Soldán T (1983) Baetidae in Mittelasien III (Ephemeroptera). Entomologische Nachrichten und Berichte 27: 266–271.

[B3] Cruz PV Nieto C Gattolliat J-L Salles FF Hamada N (2020) A cladistic insight into the higher level classification of Baetidae (Insecta: Ephemeroptera). Systematic Entomology 46(1): 44–55. 10.1111/syen.12446

[B4] Folmer O Black M Hoeh W Lutz R Vrijenhoek R (1994) DNA primers for amplification of mitochondrial cytochrome c oxidase subunit I from diverse metazoan invertebrates. Molecular Marine Biology and Biotechnology 3: 294–299. 7881515

[B5] Fujitani T Kobayashi N Hirowatari T Tanida K (2017) Morphological description of four species belonging to the genus *Nigrobaetis* (Ephemeroptera: Baetidae) from Japan. Limnology 18: 315–331. 10.1007/s10201-016-0509-4

[B6] Gattolliat J-L (2011) A new species of *Alainites* (Ephemeroptera: Baetidae) from Borneo (East Kalimantan, Indonesia). Mitteilungen der Schweizerischen entomologischen Gesellschaft, 84(3–4): 185–192. 10.5169/seals-403033

[B7] Gattolliat J-L Cavallo E Vuataz L Sartori M (2015) DNA barcoding of Corsican mayflies (Ephemeroptera) with implications on biogeography, systematics and biodiversity. Arthropod Systematics and Phylogeny 73(1): 3–18.

[B8] Jacob U (2003) *Baetis* Leach, 1815, sensu stricto oder sensu lato. Ein Beitrag zum Gattungskonzept auf der Grundlage von Artengruppen mit Bestimmungsschlüsseln. Lauterbornia 47: 59–129. [in German]

[B9] Kang SC Chang HC Yang CT (1994) A revision of the genus *Baetis* in Taiwan (Ephemeroptera, Baetidae). Journal of Taiwan Museum 47: 9–44.

[B10] Kazancı N Thomas A (1989) Compléments et corrections à la faune des Éphéméroptères du Proche-Orient: 2. *Baetiskars* n. sp. de Turquie (Ephemeroptera: Baetidae). Mitteilungen der Schweizerischen Entomologischen Gesellschaft 62: 323–327.

[B11] Kluge NJ (1997) Order mayflies – Ephemeroptera. In: Tsalolikhin SJ (Ed.) Key to freshwater invertebrates of Russia and adjacent lands, vol.3., Zoological Institute RAS, St. Petersburg, 176–220. [in Russian]

[B12] Kluge NJ Novikova EA (2014) Systematics of *Indobaetis* Müller-Liebenau & Morihara, 1982, and related implications for some other Baetidae genera (Ephemeroptera). Zootaxa 3835(2): 209–236. 10.11646/zootaxa.3835.2.3 25081445

[B13] Kluge NJ Novikova EA (2016) New tribe Labiobaetini tribus n., redefinition of *Pseudopannota* Waltz & McCafferty, 1987 and descriptions of new and little known species from Zambia and Uganda. Zootaxa 4169: 1–43. 10.11646/zootaxa.4169.1.1 27701309

[B14] Kumar S Stecher G Li M Knyaz C Tamura K (2018) MEGA X: Molecular Evolutionary Genetics Analysis across computing platforms (Version 10.0.2). Molecular Biology and Evolution 35: 1547–1549. 10.1093/molbev/msy096 29722887 PMC5967553

[B15] Lugo-Ortiz CR McCafferty WR (1999) A new genus of small Minnow Mayflies (Insecta: Ephemeroptera: Baetidae) with six new species from New Guinea and New Britain. Annales de Limnologie - International Journal of Limnology 35: 57–70. 10.1051/limn/1999013

[B16] Martynov AV Godunko RJ (2017) Mayflies of the Caucasus Mountains IV. New species of the genus *Nigrobaetis* Novikova & Kluge, 1987 (Ephemeroptera, Baetidae) from Georgia. Zootaxa 4231(1): 70–84. 10.11646/zootaxa.4231.1.4 28187550

[B17] Müller-Libenau I (1969) Revision der europäischen Arten der Gattung *Baetis* Leach, 1815. (Insecta, Ephemeroptera). Gewässer und Abwässer 48/49: 1–214.

[B18] Müller-Liebenau I Morihara DK (1982) *Indobaetis*: A New Genus of Baetidae from Sri Lanka (Insecta: Ephemeroptera) with Two New Species. Gewässer und Abwässer 68/69: 26–34.

[B19] Novikova EA Kluge NJ (1987) Systematics of the genus *Baetis* (Ephemeroptera, Baetidae), with description of new species from Middle Asia. Vestnik Zoologii 1987(4): 8–19. [in Russian]

[B20] Novikova EA Kluge NJ (1994) Mayflies of the subgenus Nigrobaetis (Ephemeroptera, Baetidae, Baetis). Entomologicheskoe Obozrenie 73(3): 623–644. [in Russian]

[B21] Sroka P (2012) Systematics and phylogeny of the West Palaearctic representatives of subfamily Baetinae (Insecta: Ephemeroptera): combined analysis of mitochondrial DNA sequences and morphology. Aquatic Insects 34(1): 23–53. 10.1080/01650424.2012.718081

[B22] Vuataz L Sartori M Wagner A Monaghan MT (2011) Toward a DNA taxonomy of alpine *Rhithrogena* (Ephemeroptera: Heptageniidae) using a mixed yule-coalescent analysis of mitochondrial and nuclear DNA. PLoS ONE 6: e19728. 10.1371/journal.pone.0019728 PMC3096624 21611178

[B23] Waltz RD McCafferty WP Thomas A (1994) Systematics of *Alainites* n. gen., *Diphetor*, *Indobaetis*, *Nigrobaetis* n. stat., and *Takobia* n. stat. (Ephemeroptera, Baetidae). Bulletin de la Société d’histoire Naturelle de Toulouse 130: 33–36.

[B24] Waltz RD McCafferty WP (1997) New generic synonymies in Baetidae (Ephemeroptera). Entomological News 108(2): 134–140.

[B25] Zrelli S Gattolliat J-L Boumaiza M Thomas A (2012) First record of *Alainitessadati* Thomas, 1994 (Ephemeroptera: Baetidae) in Tunisia, description of the larval stage and ecology. Zootaxa 3497: 60–68. 10.11646/zootaxa.3497.1.6.

